# Body surface area is positively associated with ankle-brachial index

**DOI:** 10.1177/00368504241251649

**Published:** 2024-05-23

**Authors:** Samuel Palmu, Hannu Kautiainen, Johan G. Eriksson, Harri Hakovirta, Päivi E. Korhonen

**Affiliations:** 1 Department of General Practice, 8058 University of Turku, Turku, Finland; 2 Folkhälsan Research Center, Helsinki, Finland; 3 Unit of Primary Health Care, 60650 Kuopio University Hospital, Kuopio, Finland; 4 Department of General Practice and Primary Health Care, 60652 University of Helsinki and Helsinki University Hospital, Helsinki, Finland; 5 Human Potential Translational Research programme and Department of Obstetrics and Gynecology, National University Singapore, Yong Loo Lin School of Medicine, Singapore, Singapore; 6 Singapore Institute for Clinical Sciences (SICS), Agency for Science, Technology and Research (A*STAR), Singapore, Singapore; 7 Department of Surgery, 8058 University of Turku and Southwest Finland Wellbeing Services County, Turku, Finland; 8 Department of Surgery, Satasairaala Hospital, Satakunta Wellbeing Services County, Pori, Finland; 9 Department of General Practice, 8058 University of Turku and Southwest Finland Wellbeing Services County, Turku, Finland

**Keywords:** Ankle-brachial index (ABI), peripheral artery disease, sex differences, body surface area, body size

## Abstract

**Background:**

Ankle-brachial index (ABI) measurement is a widely used diagnostic test for lower extremity artery disease. Previously, a larger body surface area (BSA) has been associated with lower blood pressure and lower 2-h post-load glucose concentrations in the oral glucose tolerance test. Our aim was to evaluate whether BSA has an impact on ABI and the prevalence of lower ABI values.

**Methods:**

ABI measurements were performed on 972 subjects aged 45 to 70 years at high cardiovascular disease (CVD) risk. Subjects with previously diagnosed kidney disease, CVD, and diabetes were excluded. Their BSA was calculated by the Mosteller formula. Study subjects were divided into ﬁve BSA levels corresponding to 12.5^th^, 25^th^, 25^th^, 25^th^, and 12.5^th^ percentiles of the total distribution. Effect modification by BSA in ABI between sexes was derived from a four-knot restricted cubic splines regression model.

**Results:**

After adjustments for age, sex, pulse pressure, glucose regulation, waist circumference, alcohol intake, smoking status, leisure-time physical activity and medication, BSA level had a positive linear relationship with ABI (*p* for linearity <0.001). When BSA was less than 2.0 m^2^, there was no difference between the sexes, but when BSA was higher than 2.0 m^2^, men had higher ABI.

**Conclusion:**

BSA shows a positive linear relationship with ABI in CVD risk subjects without manifested CVD. The difference in ABI between men and women is modified by BSA and is appreciable when BSA is larger than 2.0 m^2^.

## Introduction

Ankle-brachial index (ABI) measurement is a widely used diagnostic test for lower extremity artery disease (LEAD).^1^ Abnormal ABI is a reliable marker for cardiovascular disease (CVD) risk, generalized atherosclerosis, and all-cause mortality.^1–3^ Higher risk of CVD has also been associated with body size, e.g. short stature.^4–6^ However, the impact of body size on level of ABI is unclear. Healthy women have been reported to have lower ABI values than men which have been postulated to be a normal phenomenon associated with shorter stature.^7,8^

We have previously reported that larger body size measured by body surface area (BSA) is associated with lower blood pressure^9^ and lower 2-h post-load glucose concentrations in the oral glucose tolerance test.^10,11^ Furthermore, short stature in men has been associated with subclinical LEAD and lower ABI values in our study population.^12^

To the best of our knowledge, it is unclear whether BSA has an impact on ABI and the prevalence of lower ABI values. We hypothesized that BSA might take body size into account better than one-dimensional height, and thus, larger BSA may be associated with higher ABI values in relatively larger sized individuals.

## Methods

**Figure 1.**
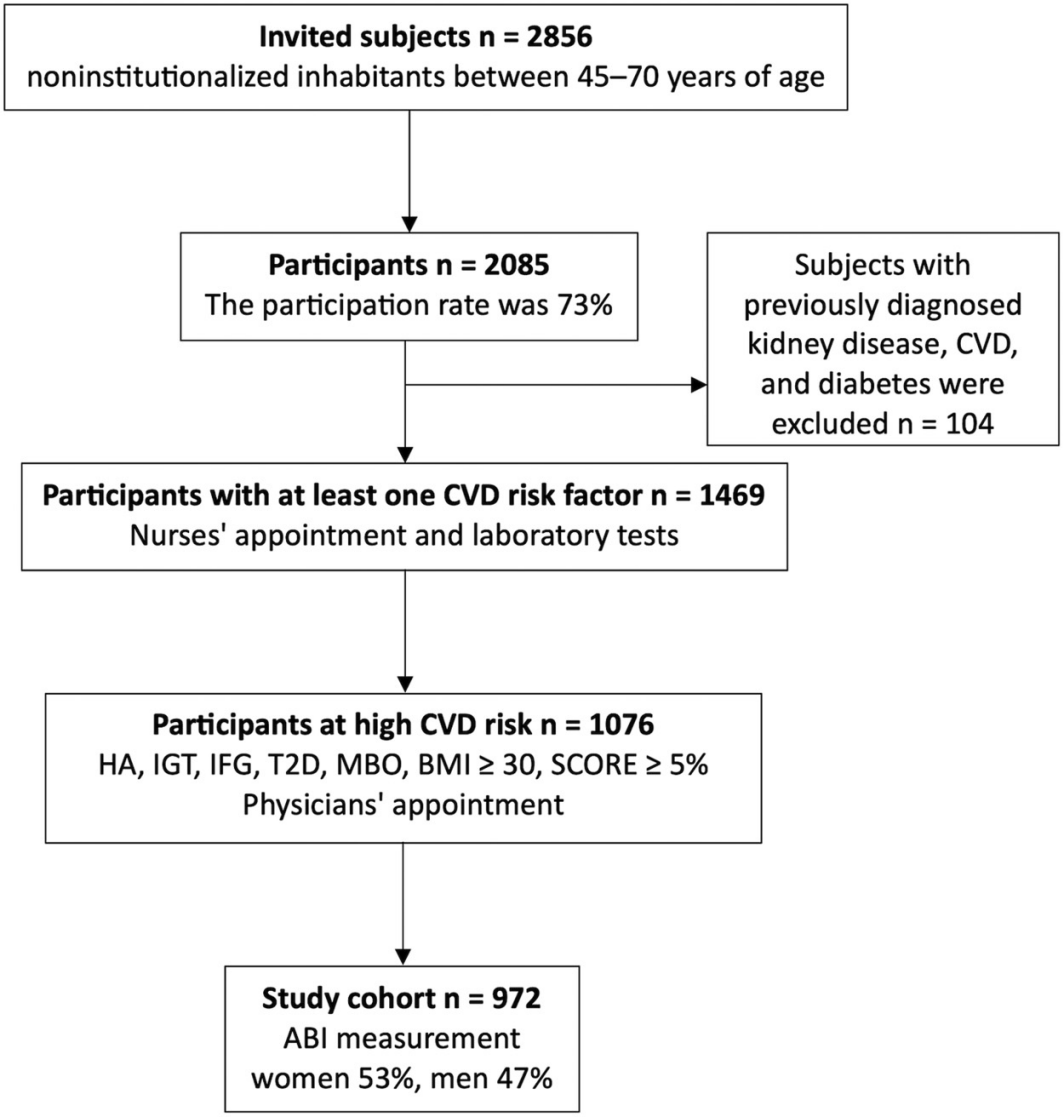
Selection of the study population. Abbreviations: ABI, ankle-brachial index; BMI, body mass index; CVD, cardiovascular disease; HA, hypertension arterialis; IFG, impaired fasting glucose; IGT, impaired glucose tolerance; MBO, metabolic syndrome; SCORE, Systematic Coronary Risk Evaluation system; T2D, type 2 diabetes.

The study population was gathered in a community-based survey, the Harmonica (Harjavalta Risk Monitoring for Cardiovascular Disease) project. Figure 1 shows the selection of the study population. The population survey was carried out in the rural town of Harjavalta from 2005 to 2006 in south-western Finland to evaluate CVD risk factors among the inhabitants. The study protocol has been described earlier in detail.^10,11,13^ An invitation letter, a validated type 2 diabetes risk assessment form (the Finnish Diabetes Risk Score questionnaire FINDRISC^14^), a cardiovascular risk factor survey, and a measuring tape for waist circumference measurement were mailed to noninstitutionalized inhabitants between 45 and 70 years of age (n = 2856). Participation and the tests were free of charge. The participation rate was 73% (2085/2856). Subjects with previously diagnosed kidney disease, CVD, and diabetes were excluded (n = 104).

A trained study nurse performed physical examination on participants who reported at least one CVD risk factor in the risk factor survey (n = 1469). Inclusion criteria included the latest measure of blood pressure ≥ 140/90 mmHg, use of antihypertensive medication, family history (parents/siblings) of coronary heart disease, stroke or myocardial infarction, history of gestational diabetes or hypertension, a FINDRISC score ≥ 12 (indicating that approximately 1 in 6 will develop type 2 diabetes within 10 years),^14^ and self-measured waist circumference ≥ 80 cm in women or ≥94 cm in men at the level of the umbilicus.

Physical examination included a measurement of height and weight in a standing position with light indoor clothing. Waist circumference was measured at the level midway between the iliac crest and the lowest rib margin. Blood pressure was measured after resting for at least 5 min using a calibrated mercury sphygmomanometer in a sitting posture. The blood pressure level was determined by calculating the mean of two readings taken at intervals of at least 2 min. BSA was calculated by the Mosteller formula [weight (kg) × height (cm)/3600]^½.^.^15^ Body mass index (BMI) was calculated as weight (kg) divided by the square of height (m^2^). The mean diastolic blood pressure (DBP) was subtracted from the mean systolic blood pressure (SBP) to calculate pulse pressure. Medical history was obtained from medical records and all subjects filled in a generic health survey.

Laboratory tests were obtained after at least 12 h of fasting. The oral glucose tolerance test was performed by measuring capillary whole blood glucose before and 2-h after ingestion of 75 g of anhydrous glucose dissolved in water. The results were converted to capillary plasma glucose values by the analyzer (HemoCue Glucose 201 + system, Ängelholm, Sweden). The WHO 1999 criteria were used to classify glucose regulation.^16^ High-density lipoprotein cholesterol (HDL-C), triglycerides, and plasma total cholesterol were measured enzymatically (Olympus AU604). Low-density lipoprotein cholesterol was calculated according to Friedewald's formula.^17^

A physician examined the high CVD risk subjects (n = 1076) who had hypertension, screen-detected impaired fasting glucose, impaired glucose tolerance, type 2 diabetes, metabolic syndrome (defined by the criteria of APT III^18^), a 10-year risk of CVD death ≥5% according to the Systematic Coronary Risk Evaluation system (SCORE),^19^ or BMI ≥ 30.0 kg/m^2^. ABI measurements were performed on 972 (517 women, 455 men) subjects. Subjects were divided into five BSA levels according to total BSA distribution percentiles: I ≤ 1.71m2 (12.5^th^), II 1.72–1.89 (25^th^), III 1.90–2.03 (25^th^), IV 2.04–2.22 (25^th^), and V > 2.22 (12.5^th^).

ABI was measured by a physician using a Doppler instrument (UltraTec® PD1v with a vascular probe of 5 MHz; Medema T/A Omega Medical Supplies Ltd, UK). An appropriate-sized blood pressure cuff was used. SBP in the brachial artery was measured in both upper arms in the antecubital fossa. SBP in the dorsal pedis artery was measured in both lower limbs. The cuff was placed just above the malleoli. The posterior tibial artery was used if the dorsal pedis pulse was not detected. The lower ankle SBP was divided by the higher brachial SBP to calculate ABI. This method to calculate ABI has been shown to identify more patients at elevated CVD risk compared to using the higher ankle pressure.^20^

The participants completed self-administrated questionnaires concerning information on alcohol consumption (Alcohol Use Disorders Identification Test, AUDIT^21^) and smoking. Leisure-time physical activity (LTPA) was classified calculating times of at least 30 min physical activity performed in a week (six or more: high; four to five: moderate; three or less: low). Use of medication was assessed.

## Statistical analysis

Data is expressed as means and standard deviations (SDs) or counts with percentages. The linearity across the three BSA levels was evaluated using the Cochran–Armitage test (Chi-square test for trend), logistic models, and analysis of variance with an appropriate contrast (orthogonal). The possible non-linear relationship between BSA and ABI values were modeled using restricted cubic splines regression models with four knots at the 5th, 35th, 65^th^, and 95th percentiles; knot locations are based on Harrell's recommended percentiles.^22^ Effect modification by BSA in ABI between sexes as the function of BSA and estimation of inflection point was derived also from a four-knot restricted cubic splines regression model. Generalized linear models were used to identify the relationship between weight and height as continuous variables and the ABI values with standardized regression coefficient Beta (β). The Beta value is a measure of how strongly the predictor variable influences the criterion variable. The Beta is measured in units of SD. Cohen's standard for Beta values above 0.10, 0.30, and 0.50 represents small, moderate, and large relationships, respectively. The models were adjusted for sex, pulse pressure, age, glucose regulation, AUDIT score, smoking status, waist circumference, LTPA, and medication, when appropriate. All analyses were performed using STATA software, version 17.0 (StataCorp LP, College Station, TX).

## Results

**Figure 2.**
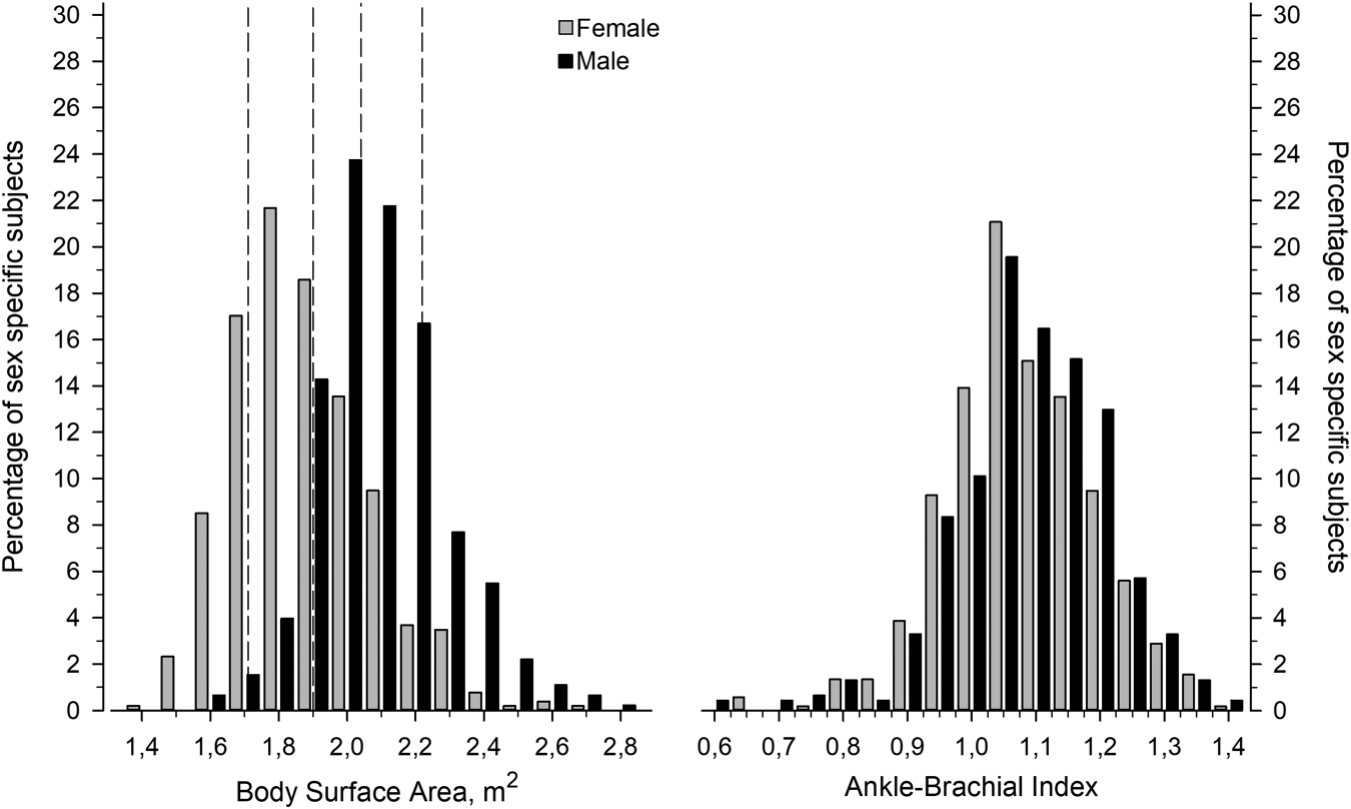
Distribution of body surface area (BSA) and ankle-brachial index (ABI) in male and female subjects. Dotted vertical lines represent BSA values divided into five level categories corresponding to 12.5^th^, 25^th^, 25^th^, 25^th^, and 12.5^th^ percentiles of the total distribution.

The study included 972 subjects at high risk of CVD. Figure 2 shows the distribution of BSA and ABI in men and women. The mean BSA was 1.98 (SD 0.22), in women 1.88 (SD 0.20) and in men 2.09 (SD 0.19), respectively. The mean ABI was 1.08 (SD 0.12), in women 1.08 (SD 0.12) and in men 1.09 (SD 0.12), respectively.

Table 1 shows the characteristics of the subjects according to the BSA levels. Subjects with lower BSA were on average older, and more often females than subjects with higher BSA. Larger body size (BSA) was positively linearly associated with anthropometric measures, DBP, fasting plasma glucose and triglyceride concentrations, prevalence of metabolic syndrome, AUDIT score and use of vasodilating drugs. BSA was negatively associated with pulse pressure and HDL-C concentration.

**Table 1.** Characteristics of the study subjects according to body surface area levels.

	Body surface area level					
	I N = 121	II N = 243	III N = 244	IV N = 243	V N = 121	*p*-value
Body surface area	≤1.71	1.72–1.89	1.90–2.03	2.04–2.22	>2.22	
Demographic:						
Number of females, n (%)	115 (95)	188 (77)	113 (46)	72 (30)	29 (24)	<0.001^a^
Age, mean (SD)	61 (7)	60 (7)	58 (7)	58 (7)	56 (6)	<0.001^a^
Weight, kg, mean (SD)	61 (5)	73 (4)	83 (4)	93 (5)	113 (12)	<0.001^a^
Height, cm, mean (SD)	158 (6)	163 (6)	169 (7)	174 (8)	179 (9)	<0.001
Waist, cm, mean (SD)	81 (7)	91 (7)	98 (7)	104 (7)	116 (10)	<0.001^a^
Body mass index, kg/m^2^, mean (SD)	24.4 (2.9)	27.5 (3.1)	29.3 (3.9)	30.8 (4.0)	35.6 (6.6)	<0.001^a^
Clinical:						
Blood pressure, mmHg, mean (SD)						
Systolic	153 (18)	149 (18)	148 (18)	148 (17)	148 (16)	0.082^a^
Diastolic	88 (8)	87 (8)	89 (9)	89 (8)	92 (9)	<0.001^a^
Pulse pressure	65 (15)	61 (14)	59 (14)	59 (13)	56 (12)	<0.001^a^
Fasting glucose, mg/dL, mean (SD)	99.3 (31.9)	100.2 (16.8)	103.4 (19.8)	103.2 (14.8)	108.3 (22.9)	<0.001^a^
Total cholesterol, mg/dL, mean (SD)	206.1 (37.1)	208.8 (39.1)	205.7 (36.3)	203.0 (34.0)	200.3 (35.2)	0.037^a^
HDL cholesterol, mg/dL, mean (SD)	66.5 (16.6)	60.7 (14.7)	57.6 (16.2)	53.8 (13.9)	48.0 (12.0)	<0.001^a^
LDL cholesterol, mg/dL, mean (SD)	120.7 (33.3)	124.9 (36.0)	123.0 (32.1)	124.1 (30.2)	123.4 (32.1)	0.76^a^
Triglycerides, mg/dL, mean (SD)	99.2 (45.2)	119.6 (54.9)	124.9 (56.7)	128.4 (67.3)	147.9 (67.3)	<0.001^a^
Metabolic syndrome present (ATPIII), n (%)	23 (19)	87 (36)	116 (48)	128 (53)	97 (78)	<0.001^a^
Glucose regulation, n (%)						<0.001^b^
Normal	70 (58)	123 (51)	111 (45)	117 (48)	54 (45)	
Impaired fasting plasma glucose	28 (23)	48 (20)	48 (20)	25 (10)	17 (14)	
Impaired glucose tolerance	17 (14)	63 (26)	70 (29)	86 (35)	37 (31)	
Type 2 diabetes	6 (5)	9 (4)	15 (6)	15 (6)	13 (11)	
Health behaviors						
Current smokers, n (%)	21 (17)	32 (13)	45 (18)	50 (21)	22 (18)	0.17^a^
AUDIT score, mean (SD)	2.7 (2.9)	4.2 (5.1)	5.8 (5.4)	5.4 (5.1)	6.0 (5.4)	<0.001^a^
LTPA, n (%)						0.079^a^
Low	20 (17)	35 (14)	30 (12)	34 (14)	13 (11)	
Moderate	81 (67)	159 (65)	179 (73)	157 (65)	78 (64)	
High	20 (17)	49 (20)	34 (14)	52 (21)	30 (25)	
Current medication, n (%)						
Vasodilators	30 (25)	66 (27)	74 (30)	73 (30)	44 (36)	0.044^a^
Beta-blockers	22 (18)	57 (23)	47 (19)	55 (23)	24 (20)	0.90^a^
Diuretics	11 (9)	24 (10)	17 (7)	30 (12)	18 (15)	0.084^a^
Statins	21 (17)	35 (14)	41 (17)	34 (14)	9 (7)	0.062^a^
ABI, n (%)						
<=0.90	9 (7)	9 (4)	16 (7)	9 (4)	6 (5)	0.47^a^
<=1.00	37 (31)	71 (29)	61 (25)	45 (19)	27 (22)	0.005^a^

a Linearity across the BSA levels.

b Differences between BSA levels.

Abbreviations: HDL, high-density lipoprotein; LDL, low-density lipoprotein; AUDIT, Alcohol Use Disorders Identification Test; LTPA, leisure-time physical activity; ABI ankle-brachial index; BSA, body surface area; SD: standard deviation.

**Figure 3.**
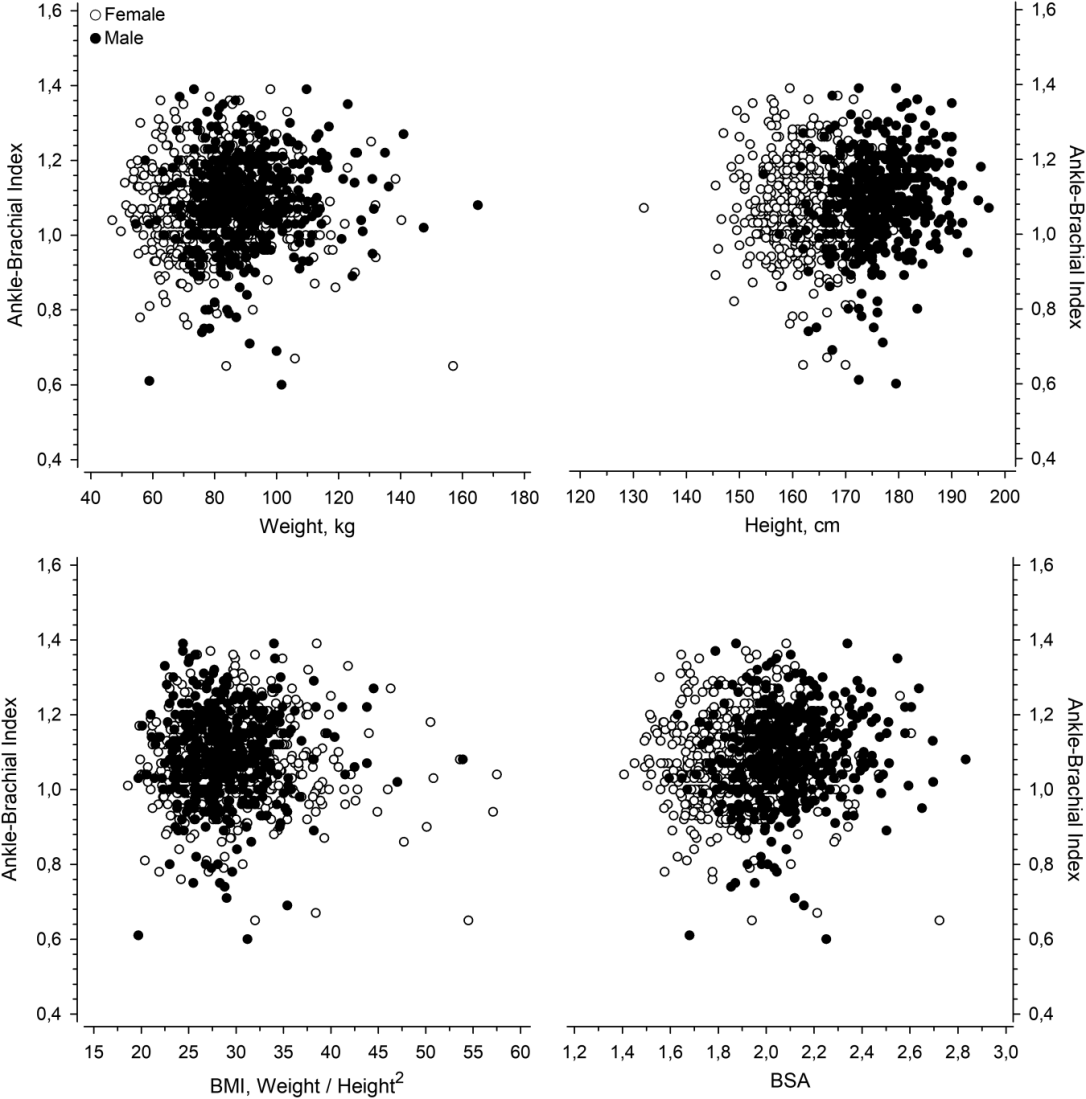
The relationships between ankle-brachial index (ABI) and weight, height, body mass index (BMI), and body surface area (BSA).

**Figure 4.**
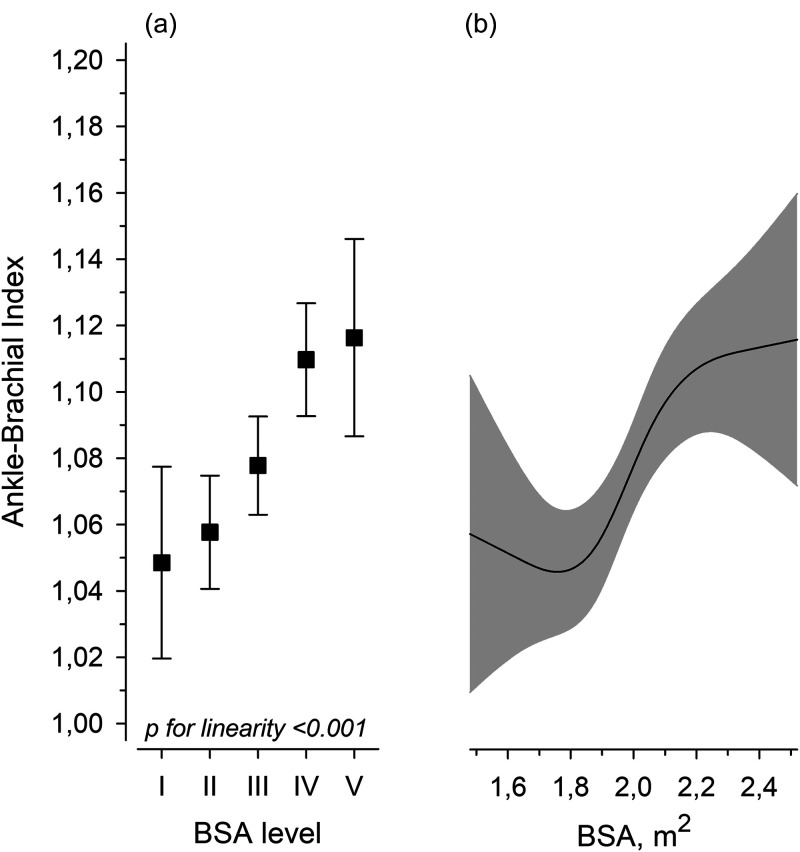
Mean ankle-brachial index (ABI) according to the levels of body surface area (BSA). Error bars are for the 95% confidence intervals. (a) The continuous values of ABI by BSA are derived from a four-knot restricted cubic splines regression model. The gray area represents the 95% confidence interval. (b) Models were adjusted for sex, pulse pressure, age, glucose regulation, waist circumference, AUDIT score, smoking status, leisure-time physical activity, and medication.

Figure 3 illustrates the relationships between ABI and weight, height, BMI, and BSA. After adjustments for age, sex, pulse pressure, glucose regulation, AUDIT score, LTPA, smoking status, and waist circumference, the BSA level showed a positive linear relationship with ABI (Figure 4a). The continuous BSA spline curve shows positive relationship with ABI (Figure 4b).

**Figure 5.**
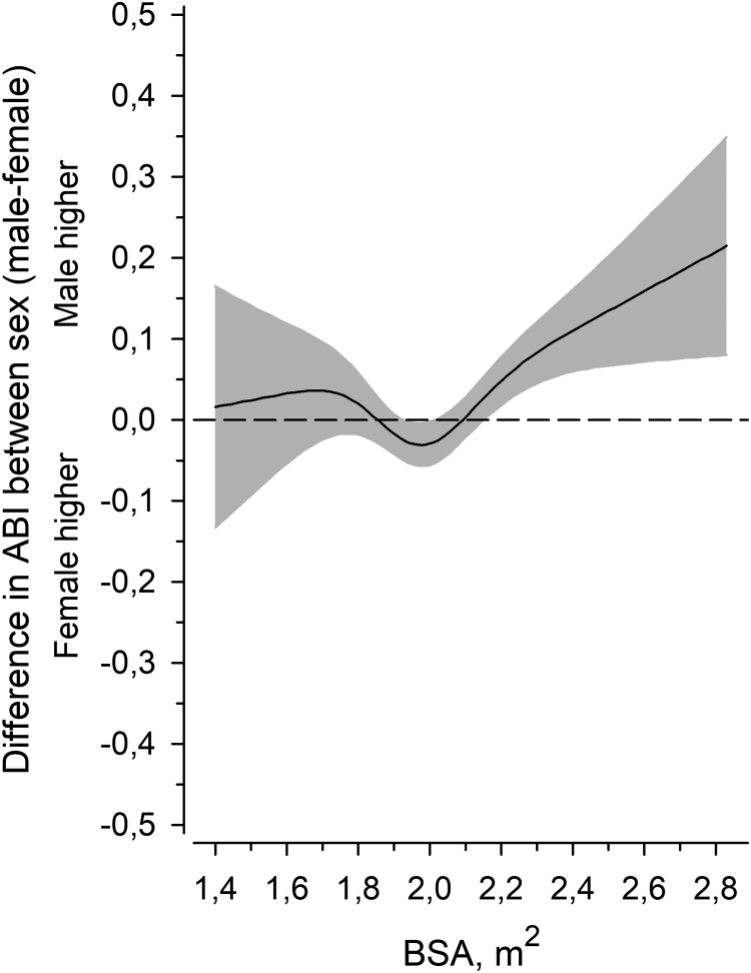
Effect modification by body size in ankle-brachial index (ABI) between sex (male–female) as the function of the body surface area (BSA) derived from a four-knot restricted cubic splines regression model. The gray area represents the 95% confidence interval. Adjusted for pulse pressure, age, glucose regulation, waist circumference, AUDIT score, smoking status, leisure-time physical activity, and cardiovascular medication.

Figure 5 shows the difference in ABI between the sexes (males minus females) as the function of BSA. When BSA was less than 2.0 m^2^ (estimation of inflection point), there was no difference between the sexes, but when BSA was higher than 2.0 m^2^, men had higher ABI.

**Figure 6.**
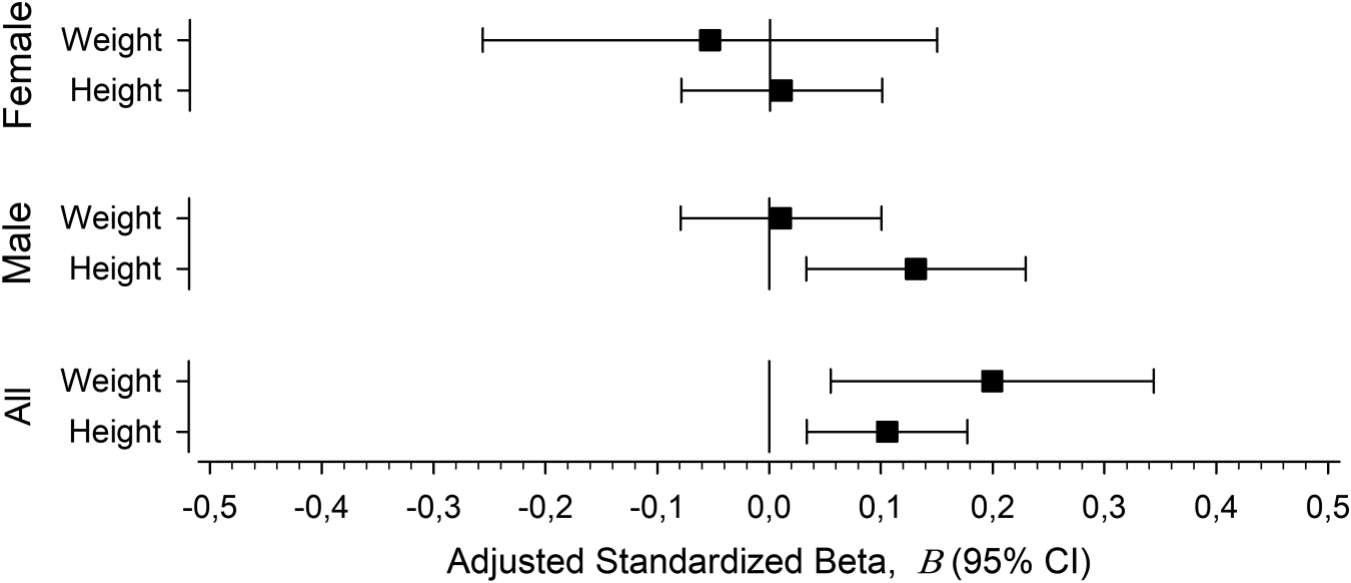
Univariate relationships between continuous body size predictive variables (height and weight) and ankle-brachial index (ABI). The adjusted standardized beta coefficients (β) with 95% confidence intervals. The models adjusted for pulse pressure, age, AUDIT score, smoking status, waist circumference, leisure-time physical activity, glucose regulation, and cardiovascular medication.

In regression analysis, height showed a positive relationship with ABI in men (Figure 6). Weight and height showed a positive relationship with ABI when both sexes were analyzed together.

## Discussion

In this study, we observed that larger BSA is associated with higher ABI in a cardiovascular risk population after adjustment for sex, pulse pressure, age, glucose regulation, AUDIT score, smoking status, waist circumference, LTPA and medication. Difference between men and women in ABI can largely be explained by effect modification by BSA. The difference is pronounced when BSA is over 2.0 m^2^.

Kapoor et al.^7^ reported that female sex is associated with lower ABI with some contribution from height. The investigators suggested that population and sex-specific percentile ABI thresholds should be utilized to diagnose LEAD in female subjects. Aboyans et al.^8^ reported in a low CVD risk population that women had lower ABI than men but the association between height and ABI was minor. It has previously been reported in our study population, that height has a positive linear association with ABI in men but not in women.^12^ Our rationale to use BSA instead of BMI is to evaluate the relationship of body size and ABI as an absolute measure taking into account both height and weight. Although BMI is a widely used measure of body size, it is a ratio of weight and square of height thus diminishing the effect of body height. We hypothesized that BSA might take body size into account better than one-dimensional height. Indeed, both body size predictive variables (height and weight) were positively associated with ABI when both sexes were analyzed together. Thus, indexing ABI values for the actual BSA of a subject might be a practical method to account for sex-specific and body size-specific differences in interpretation of ABI values.

BSA shows a positive correlation with the diameter of ascending aorta^23^ and infrarenal aortic diameter.^24^ Sandgren et al. have reported that both common femoral artery^25^ and popliteal artery^26^ diameters are positively correlated to BSA, age and sex. This may not be a result of larger lumen of vessels because of vessels in upper and lower extremity should be larger in diameter in larger persons and ABI is a ratio. Pulse wave amplitude is known to increase as a pulse travels from the aorta toward the periphery and thus ankle pressure amplification is greater and increases with body height.^8,27^

Abnormal ABI is a reliable marker for CVD risk, generalized atherosclerosis, and all-cause mortality.^1–3,8^ The relationship between short stature and higher CVD risk and mortality is well-documented.^4,5,28^ We have previously reported that larger BSA is associated with lower 2-h post-load plasma glucose concentration in an oral glucose tolerance test^10,11^ and lower blood pressure.^9^ Henriksson et al. have reported that height and serum cholesterol have negative linear relation in middle-aged men.^29^ This supports that the body size is a factor to take into account when assessing the CVD risks in epidemiological studies. Our findings suggest that BSA should be considered as a confounding factor when comparing ABI between populations with unequal body size distributions.

A strength of our study is that all ABI measurements were made by the same physician and detailed data of risk factors and medical history were gathered. The study population is quite large and represents a population at high CVD risk. A limitation of this study is the cross-sectional setting which prevent assessment of causality of relationship between BSA and ABI. Because our study is epidemiological, we did not perform any sample size or power analysis. Subjects with previously diagnosed kidney disease, CVD, and diabetes were excluded which reduces probability of media sclerotic ABI values. Proper sized cuff was used to avoid falsely high pressures due to non-compressible arteries among subject with large body habitus and large calf circumference. For logistic reasons, we used a less time-consuming, simplified method to measure ABI using SBP from dorsalis pedis artery only and posterior tibial artery was used if the dorsal pedis pulse was not detected. This could potentially lead to overestimation or underestimation of the ABI. However, the simplified method could be less time-consuming and thus practical in primary care practice to identify the subjects at high CVD risk.

## Conclusion

In conclusion, BSA shows a positive linear relationship with ABI in CVD risk subjects without manifested CVD. The difference in ABI between men and women is modified by BSA and is appreciable when BSA is larger than 2.0 m^2^. Effect modification by body size should be considered in epidemiological studies of ABI when comparing men and women. Indexing ABI values for the actual BSA of a subject might be a practical method to account for sex-specific and body size-specific differences in interpretation of ABI values.

## Supplemental Material

sj-docx-1-sci-10.1177_00368504241251649 - Supplemental material for Body surface area is positively associated with ankle-brachial index Supplemental material, sj-docx-1-sci-10.1177_00368504241251649 for Body surface area is positively associated with ankle-brachial index by Samuel Palmu, Hannu Kautiainen, Johan G. Eriksson, Harri Hakovirta and Päivi E. Korhonen in Science Progress

## Abbreviations

ABI: ankle-brachial index
AUDIT: Alcohol Use Disorders Identification Test
BMI: body mass index
BSA: body surface area
DBP: diastolic blood pressure
CVD: cardiovascular disease
FINDRISC: the Finnish Diabetes Risk Score questionnaire
HDL-C: high-density lipoprotein cholesterol
LDL-C: low-density lipoprotein cholesterol
LEAD: lower extremity artery disease
LTPA: leisure-time physical activity
SBP: systolic blood pressure
SD: standard deviation
