# Supplementary material for: Body surface area is positively associated with ankle-brachial index
Source: Sci Prog. 2024 May 23;107(2):00368504241251649. doi: 10.1177/00368504241251649 (PMC11119366; doi:10.1177/00368504241251649)
Supplement: sj-docx-1-sci-10.1177_00368504241251649 - Supplemental material for Body surface area is positively associated with ankle-brachial index [file sj-docx-1-sci-10.1177_00368504241251649.docx]

**Data Collection and Presentation Checklist:**

| **Ethical Statements (if applicable):** | |  |
| --- | --- | --- |
| **Humans** | Complete and upload the Ethics Declaration Statement you received with your decision letter. |  |
| **Animals** | Provide species, sex, strain, age, source and husbandry conditions. |  |
|  | Note if the study was blinded or not. |  |
|  | Provide a statement confirming the research was approved by the Institutional Animal Care and Use Committee. |  |
|  | Complete and upload the [ARRIVE Compliance](https://arriveguidelines.org/sites/arrive/files/ARRIVE%20Compliance%20Questionnaire%20FINAL.pdf) Questionnaire. Visit [ARRIVE](https://arriveguidelines.org/sites/arrive/files/ARRIVE%20Compliance%20Questionnaire%20FINAL.pdf) for more information. |  |
| **Reagents and Biological Materials (if applicable)** | |  |
|  | Include manufacture name, catalog number (and lot number for antibodies) for all reagents used (including fluorochromes and stains). |  |
|  | Cell lines: provide source, derivation and authentication method. |  |
| **Images (if applicable)** | |  |
| **General** | Do not introduce or remove any features in your images. Leave any blemishes. |  |
|  | If any adjustments to contrast, balance or brightness are made, they must be applied uniformly across the entire image. Any nonlinear adjustments must be disclosed in the legend. |  |
|  | Check that images are not pixelated when reasonably magnified. Images must be at 300 dpi. TIFF images are encouraged. Avoid jpegs or using PowerPoint as this will compress your images. |  |
|  | Scale bars must be included. |  |
|  | If splicing images, the borders must be marked and noted in the legend. |  |
| **Microscopy (include the following)** | Camera make and model. |  |
|  | Microscope make and model. |  |
|  | Objective magnification, type and numerical aperture. Magnification must be mentioned in the figure legend. |  |
|  | Fluorochromes and stains. They should also be mentioned in the legend. |  |
|  | Acquisition software. |  |
|  | Show all individual channels in greyscale and merged image in color (all at the same intensity). |  |
| **Western Blots** | Westerns should not be modified for contrast. The entire tonal range should be present. |  |
|  | Include **at least two** molecular weight markers, one above and one below your band of interest. [Click here to see an example](https://journals.sagepub.com/pb-assets/cmscontent/SCI/Figure_Preparation_of_Blots_and_Gels-1637348269.pdf). |  |
|  | If a blot is spliced together, you must mark the border and explain this in the legend. Splicing across different blots is not allowed. |  |
|  | If possible, normalize protein levels against total protein, not house-keeping proteins. |  |
|  | Post-translationally modified proteins (PTMs) must use total protein for normalization. |  |
|  | Provide raw blots as supplementary data. These may be combined as a single Word doc. Blots must be accurately labeled to match figures in the main doc. Include the molecular weight ladder. |  |
| **RNAi, Gene Expression, Microarrays** | At least two different siRNAs targeting different gene areas must be used. |  |
|  | At least two different control siRNAs must be used. |  |
|  | Gene expression studies cannot be presented alone without providing evidence that the changes in levels have downstream functional consequences. |  |
|  | Microarray data must include:   - - The raw data for each hybridization.   - Experimental Factors and values.   - Experimental design.   - Data processing protocols (e.g., normalization method). |  |
| **Cell culture** | At least three appropriate cell lines should be used to confirm findings. If there are fewer, add a statement explaining why only 1 or 2 were used. |  |
| **Graphs** | Bar graphs are **strongly discouraged** as **ALL** data points must be shown. Instead, consider dot plots, scatter plots or box and whisker plots. Include the appropriate error bars and average |  |
|  |  |  |
